# Using Machine Learning and Structural Neuroimaging to Detect First Episode Psychosis: Reconsidering the Evidence

**DOI:** 10.1093/schbul/sby189

**Published:** 2019-02-27

**Authors:** Sandra Vieira, Qi-yong Gong, Walter H L Pinaya, Cristina Scarpazza, Stefania Tognin, Benedicto Crespo-Facorro, Diana Tordesillas-Gutierrez, Victor Ortiz-García, Esther Setien-Suero, Floortje E Scheepers, Neeltje E M Van Haren, Tiago R Marques, Robin M Murray, Anthony David, Paola Dazzan, Philip McGuire, Andrea Mechelli

**Affiliations:** 1 Department of Psychosis Studies, Institute of Psychiatry, Psychology and Neuroscience, King’s College London, United Kingdom; 2 Huaxi MR Research Center (HMRRC), Department of Radiology, West China Hospital of Sichuan University, Chengdu, China; 3 Department of Psychoradiology, Chengdu Mental Health Center, Chengdu, China; 4 Centre of Mathematics, Computation, and Cognition, Universidade Federal do ABC, São Paulo, Brazil; 5 Department of General Psychology, University of Padova, Padova, Italy; 6 Centro Investigación Biomédica en Red de Salud Mental (CIBERSAM), Spain; 7 Department of Psychiatry, University Hospital Marqués de Valdecilla, School of Medicine, University of Cantabria-IDIVAL, Santander, Spain; 8 Neuroimaging Unit, Technological Facilities, Valdecilla Biomedical Research Institute IDIVAL, Santander, Cantabria, Spain; 9 Department of Psychiatry, University Medical Centre Utrecht, Utrecht, The Netherlands; 10 Brain Centre Rudolf Magnus, University Medical Centre Utrecht, Utrecht, The Netherlands

**Keywords:** multivariate pattern recognition/classification, psychosis, neuroimaging/multi-site

## Abstract

Despite the high level of interest in the use of machine learning (ML) and neuroimaging to detect psychosis at the individual level, the reliability of the findings is unclear due to potential methodological issues that may have inflated the existing literature. This study aimed to elucidate the extent to which the application of ML to neuroanatomical data allows detection of first episode psychosis (FEP), while putting in place methodological precautions to avoid overoptimistic results. We tested both traditional ML and an emerging approach known as deep learning (DL) using 3 feature sets of interest: (1) surface-based regional volumes and cortical thickness, (2) voxel-based gray matter volume (GMV) and (3) voxel-based cortical thickness (VBCT). To assess the reliability of the findings, we repeated all analyses in 5 independent datasets, totaling 956 participants (514 FEP and 444 within-site matched controls). The performance was assessed via nested cross-validation (CV) and cross-site CV. Accuracies ranged from 50% to 70% for surfaced-based features; from 50% to 63% for GMV; and from 51% to 68% for VBCT. The best accuracies (70%) were achieved when DL was applied to surface-based features; however, these models generalized poorly to other sites. Findings from this study suggest that, when methodological precautions are adopted to avoid overoptimistic results, detection of individuals in the early stages of psychosis is more challenging than originally thought. In light of this, we argue that the current evidence for the diagnostic value of ML and structural neuroimaging should be reconsidered toward a more cautious interpretation.

## Introduction

Over the last 3 decades, traditional mass-univariate neuroimaging approaches have revealed neuroanatomical abnormalities in individuals with psychosis.^1–5^ Because these abnormalities were detected using group-level inferences, it has not been possible to use this information to make diagnostic and treatment decisions about individual patients. Machine learning (ML) is an area of artificial intelligence that promises to overcome this issue by learning meaningful patterns from the imaging data and using this information to make predictions about unseen individuals.^6^ Several ML studies have attempted to use neuroanatomical data to distinguish patients with established schizophrenia from healthy individuals, with promising results.^7–10^ At present, however, there are two important limitations in the existing literature that limit the translational applicability of the findings in real-world clinical practice. First, given the well-established effects of illness chronicity and antipsychotic medication on brain structure,^11–15^ it is unclear to what extent classification was based on neuroanatomical changes associated with these factors rather than the onset of the illness per se. Consistent with this, both disease-stage and antipsychotic medication were identified as significant moderators in a recent meta-analysis of diagnostic biomarkers in schizophrenia.^7^ Also in line with this, Pinaya et al^16^ reported that the same ML model that was able to distinguish between patients with established schizophrenia and healthy controls (HCs) with an accuracy of 74% showed poor generalizability (56%) when applied to a cohort of individuals with first episode psychosis (FEP). Taken collectively, these findings suggest that representations learned from patients with established schizophrenia may not be applicable to individuals with a first episode of the illness. Second, the clinical utility of any ML-based diagnostic tool for detecting patients with an established illness is likely to be very limited; in contrast, detecting the initial stages of an illness, when diagnosis may be uncertain and treatment is yet to be decided, is likely to have much greater clinical utility.

So far only a limited number of studies have applied ML to neuroanatomical data in the initial stages of the illness when the effects of illness chronicity and antipsychotic medication are minimal. These studies have produced inconsistent results, including poor (eg, 51% in Winterburn et al^17^), modest (eg, 63% in Pettersson-Yeo et al^18^), and good (eg, 86% in Borgwardt et al^19^ or 85% in Xiao et al^20^) accuracies. There are a number of possible reasons for such inconsistency. First, most of the studies used small samples (*N* ≤50) (see Kambeitz et al^7^ for a meta-analysis), which have been shown to yield unstable results.^21,22^ Second, the vast majority of studies used data from a single site, and as such may have generated results that were specific to the characteristic of the local sample rather than the illness per se. Third, a series of recent articles have highlighted potential methodological issues that may have caused inflated results in some of the published studies.^9,17,22–25^ These issues include, eg, (1) failure to use a nested cross-validation (CV) framework to avoid *knowledge-leakage* between training and test sets; (2) failure to perform feature transformation and/or selection within a rigorous CV framework resulting in so-called “double dipping”; (3) publication bias leading to an overrepresentation of positive findings, especially in studies with small samples and (4) failure to test performance on additional independent samples. Also, we note that all studies have employed traditional “shallow” ML techniques, such as support vector machine and logistic regression. The intuitiveness of such techniques has made them very popular in neuroimaging studies of psychiatric and neurological disease. Deep learning (DL) is an alternative type of ML, which has been gaining considerable attention in clinical neuroimaging.^9,16,23,26^ Contrary to traditional ML, where the immediate input data are used to extract patterns (hence the term “shallow”), DL learns complex latent features of brain structure through consecutive nonlinear transformations (hence the term “deep”), which are then used for classification. Given its ability to learn more intricate and abstract patterns, DL might be particularly suitable to detect the subtle and heterogeneous neuroanatomical abnormalities characteristic of the early stages of psychosis.^1,27,28^

This study aims to elucidate the extent to which the application of ML to neuroanatomical data allows distinction between patients with FEP and HCs at the individual level. To overcome the limitations of previous studies, we used a total of 5 datasets from different sites, each with a sample size above the recommended threshold for a stable performance,^21^ and employed both shallow and deep ML techniques. In addition, following a series of recent articles highlighting potential methodological issues in the existing literature,^9,17,22–25^ we put in place a series of precautions to minimize the risk of overfitting. On the basis of previous studies, we hypothesize that (1) FEP and HC will be classified with statistically significant performances ranging between 70% and 80%^7^ and (2) DL will perform better than traditional shallow approaches.^26^

## Methods

### Subjects

Participants were recruited as part as 5 independent studies carried out in multiple sites, all of which have been previously published:

- Site 1: Chengdu, China^29^
- Site 2: London, England (Genetic and Psychosis study^30^)
- Sites 3 and 4: Santander A and B, Spain (Programa Asistencial Fases Iniciales de Psicosis (First Episode Psychosis Clinical Program) study^31^)
- Site 5: Utrecht, The Netherlands (Genetic Risk and Outcome of Psychosis study^32^)

All patients were experiencing their first psychotic episode, defined as the first manifestation of psychotic symptoms meeting criteria for a psychotic disorder, as specified by the DSM-IV^33^ or ICD-10^34^. The demographic and clinical characteristics, including duration of illness, are reported in table 1. For information on recruitment criteria, see supplementary material.

**Table 1.** Demographic and Clinical Characteristics for FEP and HC for Each Site

		Chengdu, China (N = 222)		London, England (N = 142)		Santander A, Spain (N = 220)		Santander B, Spain (N = 210)		Utrecht, The Netherlands (N = 162)	
		HC	FEP	HC	FEP	HC	FEP	HC	FEP	HC	FEP
*n*		111	111	71	71	110	110	70	140	81	81
Gender (%)	M	51 (46)	51 (46)	36 (51)	36 (51)	68 (62)	68 (62)	45 (64)	90 (64)	64 (79)	64 (79)
	F	61 (54)	61 (54)	35 (49)	35 (49)	42 (38)	42 (38)	25 (46)	50 (46)	17 (21)	17 (21)
		χ2 = ns		χ2 = ns		χ2 = ns		χ2 = ns		χ2 = ns	
Age M (SD)		27.2 (7.3)	25.7 (8.1)	26.8 (7.1)	26.4 (6.2)	29.7 (7.8)	28.5 (8.6)	27.3 (7.5)	28.3 (7.6)	26.9 (8.0)	25.2 (5.9)
		*t* = ns		*t* = ns		*t* = ns		*t* = ns		*t* = ns	
TIV (L) M (SD)		1.5 (0.1)	1.5 (0.2)	1.5 (0.2)	1.5 (0.2)	1.5 (0.1)	1.4 (0.2)	1.5 (0.1)	1.5 (0.1)	1.6 (0.1)	1.5 (0.2)
		*t* = ns		*t* = ns		*t* = ns		*t* = ns		*t* = ns	
Positive symptoms M (SD)		—	24.6 (6.6)^a^	—	13.9 (5.5)^a^	—	14.7 (4.6)^b^	—	14.4 (4.1)^b^	—	15.9 (6.3)^a^
Negative symptoms M (SD)		—	18.2 (7.7)^a^	—	16.0 (6.0)^a^	—	6.3 (4.6)^c^	—	6.1 (5.0)^c^	—	16.2 (6.9)^a^
Duration of illness (years) Med (IQR)		—	0.3 (1.1)	—	1.1 (0.3)	—	0.3 (0.7)	—	0.3 (0.9)	—	0.6 (1.0)

*Note*: TIV, total intracranial volume; L, liters; M, male; F, female; FEP, first episode psychosis; HC, healthy controls, SD, standard deviation; Med, median; IQR, interquartile range.

^a^PANSS: Positive and Negative Symptoms Scale.

^b^SAPS: Scale for the Assessment of Negative Symptoms.

^c^SANS: Scale for the Assessment of Negative Symptoms.

ns: *P* > .05

### MRI Data Acquisition and Preprocessing

High-resolution three-dimensional T1-weighted images were acquired independently at each site (supplementary table 2). From each image, 3 types of data features were extracted (see supplementary material):

- Voxel-based gray matter volume (GMV): whole-brain voxel-wise estimate of the local density of gray matter (GM) in a given voxel region^35^
- Voxel-based cortical thickness (VBCT): cortical thickness maps in which each voxel in the GM is assigned a thickness value^36,37^
- Surfaced-based regional volumes and cortical thickness: volume and thickness of predefined cortical and subcortical regions extracted with FreeSurfer^38^

### Statistical Analysis

#### Demographic and Clinical Variables

Differences in age, gender, and total intracranial volume between FEP and HCs were examined using an independent-samples *t*-test and chi-square test, as implemented in the Statistical Package for the Social Sciences 24.0 (SPSS 24.0).

#### Group-Level Comparisons

For completeness, a standard group-level analysis was also carried out for each site and type of feature set separately. See supplementary material sections 1.4.1. and 2.1 for methods and results, respectively.

#### Multivariate Pattern Recognition Analysis

##### Dimensionality Reduction: Principal Component Analysis 

Principal component analysis (PCA) was used to reduce the number of voxels of the GMV and VBCT maps (see supplementary material).

##### Classifiers 

**Fig. 1.**
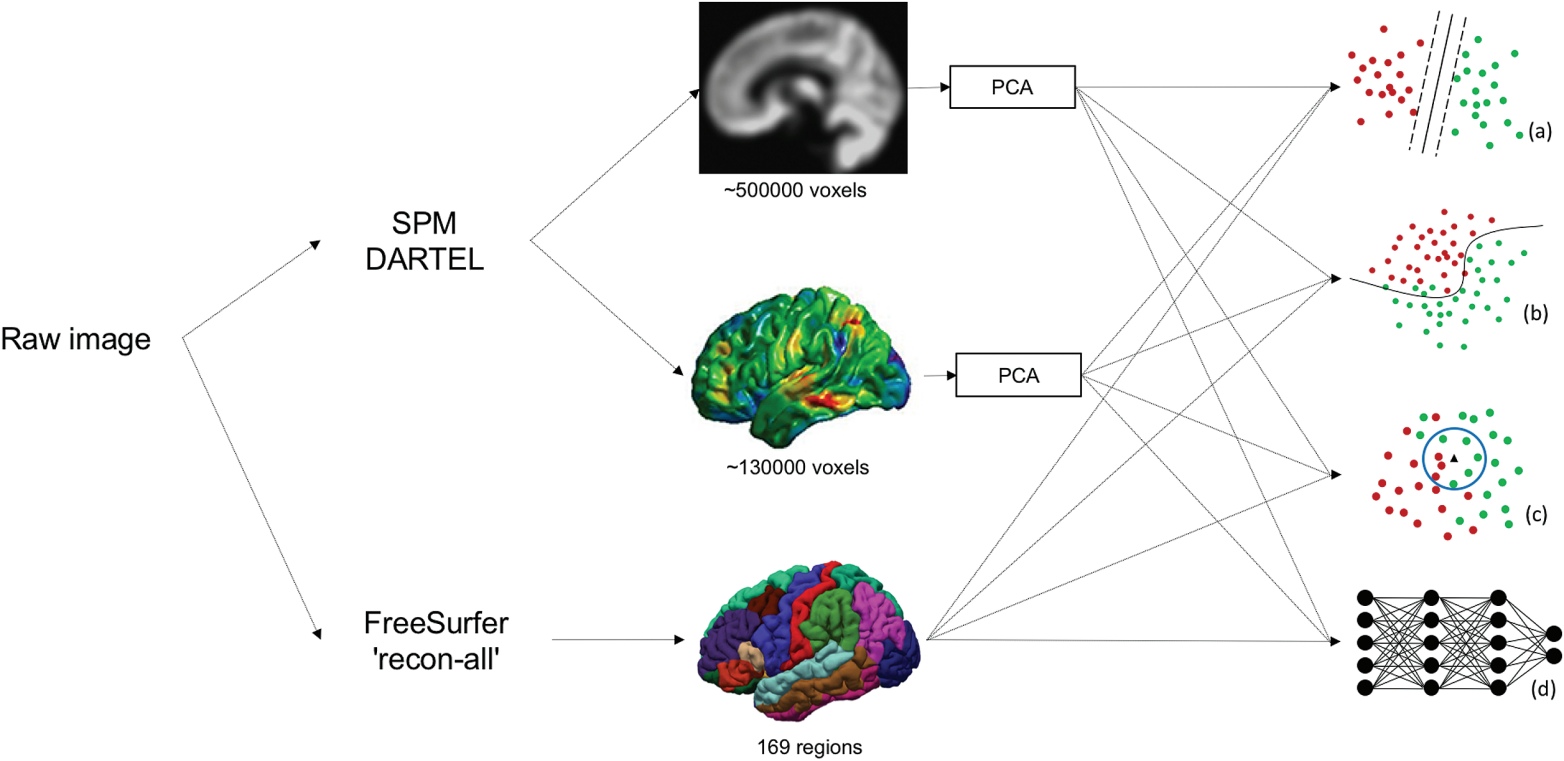
Three features were extracted from each image: GMV, VBCT, and FreeSurfer surface-based regional volumes and cortical thickness. The dimensionality of GMV and VBCT was reduced through PCA. The resulting features were analyzed with four classifiers: (a) SVM, (b) LR, (c) KNN and (d) DNN. GMV, gray matter volume; VBCT, voxel-based cortical thickness; PCA, principal component analysis; SVM, support vector machine; KNN, k-nearest neighbors; LR, logistic regression; DNN, deep neural network.

Four methods were used for classification: k-nearest neighbors (KNN), logistic regression (LR), support vector machine (SVM) and deep neural networks (DNN) (see supplementary material). These methods were chosen based on their increasing order of complexity (KNN is a straightforward algorithm, whereas DL can be more powerful at the expense of transparency), popularity (SVM and LR are among the most ML techniques used in previous studies), and novelty (DL has yielded promising results in psychiatric neuroimaging but is yet to be applied to FEP) (figure 1).

KNN: non-parametric method that uses the distance between data points to make new predictions by assigning unseen data to the same class to which the closest data points belong to^39^.

LR: regression model applied to one dependent categorical variable implemented via elastic net, a regularized regression that combines the regularizations L1 and L2 penalties of Least Absolute Shrinkage and Selection Operator (LASSO) and ridge regression, respectively, to avoid overfitting.^40^

SVM: method that estimates a hyperplane with an optimum margin that best separates two classes, determined by the maximum distance from any data point. Once defined, this hyperplane is used to classify unseen data.^41,42^

DNN: multi-layered fully connected networks in which higher-level features are learned as a nonlinear combination of lower-level features, allowing the extraction of complex and abstract patterns.^43^

##### Model Training and Testing

###### Within-site classification

**Fig. 2.**
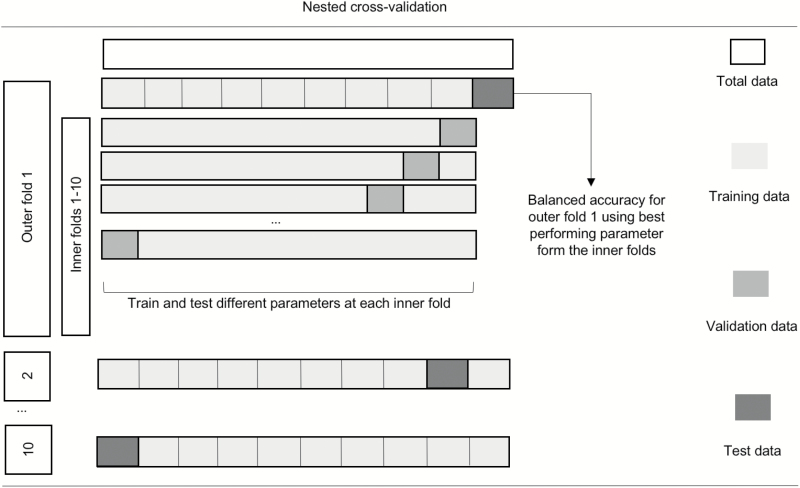
Schematic representation of nested CV. Nested CV involves a secondary inner CV loop using the training data from the primary outer CV split, where different sets of hyperparameters are tested (eg, different values for the C parameter for SVM). The best-performing hyperparameters among the 10 inner folds are then used to train a model in the whole training set defined by the outer loop. This model is then tested using the test set of the outer loop. The final performance is estimated by averaging accuracies in the test set across all 10 outer folds. CV, cross-validation; SVM, support vector machine.

All models were assessed through a nested 10-fold stratified CV framework (figure 2) to ensure that the data for hyperparameter tuning and the data to test the algorithm were strictly independent. A 10-fold CV was chosen as a trade-off between bias, variance, and the demanding computational resources required to run DNN.

###### Cross-site classification

The best site-level model was further tested in each one of the remaining independent samples. All 10 instances trained during the CV were used to classify the participants from all the remaining sites separately. The resulting ensemble of models predicted the class of each participant using the soft voting method, where the class label was defined by the average of the 10 predicted probabilities.

###### Performance Measures 

Balanced accuracy, sensitivity, and specificity were chosen as the performance metrics. Statistical significance of the balanced accuracy was determined by permutation testing with 1000 permutations (see supplementary material).

###### Effect of Antipsychotic Medication and Psychotic Symptoms

To examine whether antipsychotic medication or psychotic symptoms contributed to the classifiers’ performance, chlorpromazine equivalents and positive and negative psychotic symptoms were regressed against the predicted labels using an logistic regression (see supplementary material for details).

### Results

#### Sociodemographic and Clinical Parameters

No statistically significant differences were identified between patients and controls for age, gender, or total GMV at each site (table 1).

#### Single-Subject Classification

##### Can We Detect FEP at the Individual Level?

Balanced accuracy, sensitivity, specificity, and statistical significance for each feature set of interest and site are presented in table 2 (for a visual display of the accuracies and standard deviations see supplementary figure 3 in the supplementary material). Overall, results were poor to modest across all types of feature sets and sites, although the site with the smallest sample size (site 2) showed the lowest performance consistently across all feature sets. Overall, regression analyses examining the effect of antipsychotic medication and psychotic symptoms on the performance of each classifier did not show a significant effect (see supplementary material).

**Table 2.** Accuracies (Sensitivity/Specificity) for Each Feature Set and Algorithm Across All Sites Using Nested 10-fold Stratified Cross-Validation. The Classifier Yielding the Best Balanced Accuracy Is Highlighted in Bold for Each Site

		Regional volumes and cortical thickness	GMV	VBCT
Site 1 Chengdu, China	KNN	60.7^**^ (74.3/47.1)	**60.7** ^**^ **(49.5/71.9)**	62.1^**^ (72.1/52.1)
	LR	61.9^**^ (64.9/58.9)	60.1^**^ (62.9/58.6)	**67.2** ^**^ **(65.8/68.5)**
	SVM	61.3^**^ (66.4/56.2)	**60.7** ^**^ **(63.0/58.5)**	52.7* (24.6/97.3)
	DNN	**70.5** ^**^ **(72.2/68.8)**	57.7^**^ (59.5/56.0)	66.4^**^ (63.9/68.3)
Site 2 London, England	KNN	56.7 (50.9/62.5)	43.9 (33.6/54.3)	53.5 (38.4/68.6)
	LR	51.6 (45.0/58.2)	51.9 (53.8/50.0)	**61.6** ^**^ **(63.2/60.0)**
	SVM	45.9 (49.3/42.5)	**53.9 (53.4/54.3)**	51.0 (96.3/5.7)
	DNN	**58.8** ^*^ **(49.5/68.0)**	40.8 (47.4/34.3)	53.4 (52.4/55.3)
Site 3 Santander A, Spain	KNN	59.6^**^ (45.5/73.6)	50.5 (31.8/69.1)	58.0* (50.0/66.4)
	LR	58.6* (58.2/59.1)	63.2^**^ (63.6/62.7)	59.1* (58.2/60.0)
	SVM	60.5^**^ (61.8/59.1)	**65.9** ^**^ **(68.2/63.6)**	51.8* (90.9/12.7)
	DNN	**70.2** ^**^ **(70.0/70.4)**	50.2 (52.7/63.6)	**59.6 (60.0/59.1)**
Site 4 Santander B, Spain	KNN	56.6* (91.8/21.4)	58.9^**^ (70.7/47.1)	59.5* (67.7/51.1)
	LR	54.8 (73.9/35.7)	59.6^**^ (57.8/61.4)	**62.6** ^**^ **(56.8/62.4)**
	SVM	56.0 (65.0/47.1)	57.4* (71.9/42.9)	58.4* (71.9/52.9)
	DNN	**62.0** ^**^ **(76.8/47.1)**	59.3* **(81.4/37.1)**	58.8^**^ (62.4/53.1)
Site 5 Utrecht, The Netherlands	KNN	52.7 (53.6/51.8)	54.5 (33.8/75.3)	52.2 (36.5/67.9)
	LR	58.5* (61.7/55.4)	61.3^**^ (56.8/65.7)	**60.5** ^**^ **(60.6/60.4)**
	SVM	**60.7** ^**^ **(59.7/61.7)**	**62.4** ^**^ **(63.1/61.8)**	56.3 (51.2/61.4)
	DNN	54.9 (59.2/51.8)	58.0^**^ (58.1/57.9)	60.1^**^ (56.1/64.2)

*Note*: SVM, support vector machine; LR, logistic regression, KNN, k-nearest neighbors; DNN, deep neural network; GMV, voxel-based gray matter volume; VBCT, voxel-based cortical thickness.

*P < .05; ^**^P < .01.

##### What Are the Most Effective Type of Feature Set?

There was no clear effect of type of feature set across sites. However, it can be seen that surface-based regional data tended to yield higher accuracies, especially when analyzed with DNN.

##### Can We Generalize the Results From One Site to the Others?

The best performances were achieved by two DNN models at sites 1 and 3 using regional volumes and cortical thickness, with 70.5% and 70.2%, respectively. However, both models generalized poorly when tested on the remaining sites: specifically, the DNN model from site 1 achieved accuracies (sensitivity/specificity) of 52.1% (56.3%/47.9%), 61.1% (70.0%/52.7%), 52.1% (65.7%/38.6%), and 50.0% (48.3%/51.7%) when applied to sites 2 through 5, respectively; whereas the DNN model from site 3 achieved accuracies of 52.2% (96.5%/8.4%), 49.2% (83.5%/33.4%), 55.1% (70.1%/40.0%), and 51.0% (67.5%/34.6%) when applied to sites 1, 2, 4 and 5, respectively. To examine the possibility that poor generalizability was due to site differences, the same DNN model was applied to the total data with the 5 sites added as additional features. Features weights were then investigated to determine the importance of site. Results showed that out of the 174 features, the weights for site 1, 2, 3, 4, and 5 ranked 110, 150, 108, 71, and 112, respectively.

### Discussion

In the last few years, there has been increasing interest in the translational potential of ML approaches in psychosis. As the field matures, there is emerging skepticism about replicability and generalizability, which has led to recent calls for greater caution in the interpretation of the findings.^9,17,22,23,25^ This study aimed to elucidate the extent to which the application of ML to neuroanatomical data allows detection of individuals at the early stages of psychosis when the effects of illness chronicity and antipsychotic medication are minimal. To overcome the limitations of the existing literature, we used 5 independent datasets and put in place a series of methodological precautions to avoid overoptimistic results. Contrary to expectation, the performances of all methodological approaches tested were poor to modest across all sites. Later we discuss some of the main aspects that emerge from our investigation, including sample size, full independence of training and test data, cross-site generalizability, and testing multiple pipelines. We conclude the discussion by considering possible future directions.

#### Sample Size, Homogeneity, and Publication Bias

**Fig. 3.**
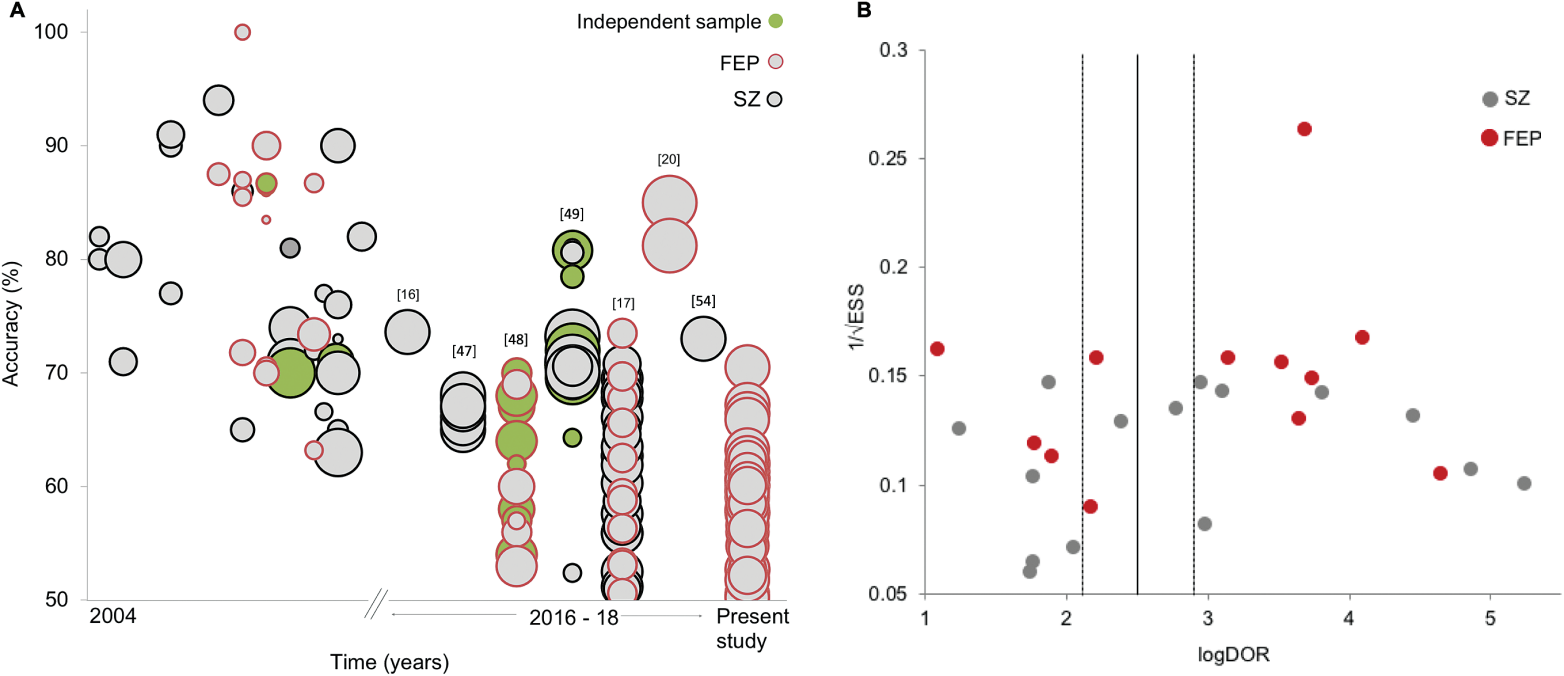
(**A**) Accuracy of diagnostic sMRI ML studies over time and sample size (circle increases with sample size). From the first study until 2015, the vast majority of studies reported accuracies ranging between 70% and 100%; from 2016, however, performances have dropped overall with accuracies ranging between chance-level and 85%. (**B**) Funnel plot for sMRI studies in schizophrenia and FEP showing the distribution of individual studies according to their sample size (1/√ESS) and effect size (log diagnostic odds ratio). The plot revealed statistically significant asymmetric distribution around the main effect of sMRI studies (*P* = .013), indicating a bias favoring higher effect sizes. sMRI, structural MRI; ML, machine learning; FEP, first episode psychosis.

A possible explanation for why our accuracies are lower than those reported in the existing literature is that some of the previous studies may have reported overoptimistic results due to the use of fairly small sample sizes. To illustrate this possibility, we tested for an association between sample size and classification accuracy across studies using ML and structural MRI (sMRI) in the existing literature (see supplementary material). Unsurprisingly, we found a moderate negative association for studies that examined established schizophrenia (*r* = −.41) and FEP (*r* = −.59; after excluding Xiao et al,^20^ which was a clear outlier; figure 3A). This is consistent with the notion that some of the previous studies may have reported overoptimistic accuracies due to the use of inadequate sample size.

There are at least two possible ways in which inadequate sample size can lead to an inflated estimation of the accuracy of an algorithm, including sample homogeneity and publication bias.^22,25^ First, smaller samples tend to be more homogeneous, making it easier for an algorithm to learn shared abnormalities in patients relative to controls and resulting in higher accuracies. In contrast, larger samples tend to be more heterogeneous due to the loosening of inclusion criteria; in this case, it may be more challenging to find a shared pattern of abnormalities resulting in lower performances. This inverse relationship between sample size and accuracy was not observed in our investigation; however, this might be explained by the fact that there was not sufficient variability in sample size across our five datasets. Second, smaller samples tend to be unstable and thus yield underestimated as well as overestimated accuracies.^21,44^ This may, in turn, lead to publication bias, with overestimated accuracies being more likely to be published. In their meta-analysis of ML studies of schizophrenia, Kambeitz et al^7^ reported that no publication bias was evident when all studies—including sMRI, functional magnetic resonance imaging, and DTI—were examined together. To test for publication bias in sMRI studies, we repeated the same statistical analysis focusing on this modality (see supplementary material). This revealed a statistically significant asymmetry in the funnel plot of published studies, indicating the presence of publication bias (figure 3B). This is in line with emerging concerns about possible overrepresentation of inflated performances in the literature.^17,22,23,25^

#### Full Independence of Training and Testing Set Data

Following recent recommendations on how to overcome methodological issues that may have led to initial inflated results,^9,23,25^ we adopted two important methodological precautions. First, the use of simple CV, in which the same test data are used to both tune model hyperparameters and evaluate its performance, has been criticized as it almost certainly leads to inflated performances.^45,46^ In the present investigation, algorithms were trained and tested via nested CV. This ensured that the test set remained fully independent from the training set, with only the latter being used to optimize model parameters. Second, implementing feature selection in a 2-step approach, where, eg, univariate tests (eg, *t*-test) are applied in the whole sample and only the statistically significant features are used for classification, is likely to result in overoptimistic performances as features are chosen based their performance on data that should be completely independent for testing the classifier. In the present investigation, therefore, transformations to the data, such as feature selection, were implemented within the CV framework, ie, parameters were derived from the training data only and subsequently applied to the test set. The adoption of these methodological precautions, aimed at ensuring full independence between training and test data, might explain the fact that accuracies in the present investigation were lower than expected.

#### Cross-Site Generalizability

The use of independent samples to develop and validate an algorithm is a critical requirement if the ultimate aim is to develop flexible ML-based tools that could be used in a clinical setting.^23,25^ However, only a minority of studies have attempted to do this, eg,^22,47,48^, and most of them have reported considerably lower performances in the independent sample. In the present investigation, the highest accuracies—obtained using specific combinations of dataset, type of feature set and algorithm—were 70% (in sites 1 and 3 with surface-based regional features and DNN); this performance would appear to be in line with previous similar studies. However, selectively reporting these accuracies from our wider set of results would have portrayed a distorted picture of the potential of ML to detect the initial stages of psychosis at the individual level.^24^ This is especially true since after testing these two models in independent datasets, their performance did not hold up, indicating low cross-site generalizability. Such low cross-site generalizability could be due to site-related differences in scanning parameters, cultural interpretation of diagnostic criteria, and ethnicity; therefore, it might be possible to achieve higher cross-site generalizability by combining samples that are homogenous with respect to these variables. Nevertheless, our current results indicate that algorithms developed using data from a specific centre do not perform well when applied to data from other centers, and thus have limited clinical applicability.

#### Testing Multiple Pipelines

Because existing studies tend to differ with respect to several methodological aspects, at present, it is difficult to say which pipeline is optimal for detecting FEP.^47^ Multi-pipeline studies have therefore been proposed as a useful way to disentangle what aspects works best.^23^ Importantly, this approach may also help build more generalizable models, as the development of a bespoke, and possibility overfitted, pipeline to a local sample is less likely to occur. Consistent with this, Salvador et al^47^ tested the performance of a range of ML approaches in different types anatomical features extracted from patients with schizophrenia and controls, and reported lower accuracies (66%–68%) compared to previous similar studies using a single pipeline. Winterburn et al^17^ also used multiple pipelines in FEP and reported poor to modest accuracies, ranging from 51% to 73%. Taken collectively, evidence from these studies, including our own, suggest that when features are not manually carved to fit one algorithm applied to one specific small dataset, performance tends to drop. This can be seen in figure 3A where two generations of studies emerge: initially, there were mostly small single-site, single-feature, and single-algorithm high-performance studies; more recently the use of (1) larger samples,^16,47,20,54^ (2) multicentre studies,^48,49^ (3) assessment of different algorithms and/or features in one/several site(s),^17,47^ or (4) independent sample testing^48,49^ are reshaping the original, and possibly overinflated, enthusiasm with more realistic performances.

#### What Next for ML-sMRI Studies of Psychiatric Disease?

Unlike group-level analysis, where larger samples lead to increased chance of detecting a statistically significant result (even with a small effect size), in ML larger samples do not necessarily equate to better results; instead, these tend to lead to lower accuracies due to increased heterogeneity.^22,28^ Despite this challenge, larger samples are likely to be more representative of the illness, less likely to overfit and thus carry more translational potential. Future ML studies will have to address this issue to overcome the increasingly apparent bottleneck in the performance that is arising with larger sample sizes (figure 3A). A possible way of doing so could be to use normative models, where an individual is mapped against a normative model that should encompass the heterogeneity characteristic of the normal population. Here, illness is considered an extreme case within a normal range, which is likely to be a more ecologically valid approach than the traditional case–control paradigm.^50,51^

Greater methodological standardization based on “good-practice recommendations” could also help disentangle the current conflicting evidence. For example, guidelines for minimum sample size such as the threshold (*n* > 130) proposed by Nieuwenhuis et al^21^ are a good start. The need for independent sample testing has also been widely acknowledged as an essential step toward generalizability^23,25^; however, even the most recent studies do not always perform this. Moving forward, this type of generalizability test is likely to become a gold standard for ML diagnostic studies. More transparency in the implementation of ML is also needed. Several studies do not provide enough information about how the algorithm was trained and tested.^23,28,52^ This hinders a thorough assessment of the validity of the study as well as its replicability. Finally, it should be noted that, even if sMRI was able to distinguish between patients with FEP and disease-free individuals with high levels of accuracy, this would be of limited clinical utility. This is because, from a clinical translation perspective, the real challenge is not to distinguish between patients and disease-free individuals, but to develop biological tests that could be used to choose between alternative diagnoses and optimize treatment.^52^

### Conclusion

The present investigation attempted to overcome the limitations of the existing literature using a number of strategies. First, we studied patients with FEP in which the effects of antipsychotic medication and illness chronicity are likely to be minimal. Second, the sample size of each of our 5 datasets was greater than the recommended threshold for achieving a stable performance in ML–sMRI studies.^21^ Third, critical methodological precautions (eg, nested CV and appropriate use of feature selection) were adopted to ensure an unbiased assessment of performance. Fourth, we systematically assessed the performance of a range of algorithms and features across several datasets, thereby minimizing the possibility of developing a bespoke and likely overfitted model to a single site. Fifth, we assessed the cross-site generalizability of the best models at the single-site level. Our findings suggest that the use of ML and sMRI allows detection of FEP at the individual level with relatively modest accuracies—lower than what was expected based on previous studies and much lower than what would be required for clinical translation. We speculate that some of the previous results may have been over-optimistic due to a combination of small sample sizes, less-than-rigorous methodologies, and possible publication bias and argue that the current evidence for the diagnostic value of ML and structural neuroimaging should be reconsidered toward a more cautious interpretation.

Over the past few years, the number of ML studies in psychosis has been increasing rapidly.^52^ As larger samples and more powerful computational resources become available, this momentum is likely to continue to grow over the coming years.^53^ Therefore, it is important for the research community to be aware of the challenges and limitations of applying ML to psychosis such as the several potential “distortion” of the findings along the ML pipeline, as discussed in a recent review.^52^ In light of these challenges and limitations, the extent to which the application of ML in psychosis will lead to a more valid construct of the illness remains an open question. We encourage researchers to continue pursuing the integration of ML and neuroimaging, while exercising caution to avoid inflated results and ultimately a distorted view of the potential of this approach in psychiatric neuroimaging.

## Funding

This work was supported by the European Commission (PSYSCAN—Translating neuroimaging findings from research into clinical practice; 603196 to P.M.); International Cooperation and Exchange of the National Natural Science Foundation of China (81220108013 to Q.G. and A.M.); Wellcome Trust’s Innovator Award (208519/Z/17/Z to A.M.); Foundation for Science and Technology (SFRH/BD/103907/2014 to S.V.), and São Paulo Research Foundation (FAPESP) (Brazil; 2013/05168-7 to W.H.L.P.). The authors have declared that there are no conflicts of interest in relation to the subject of this study.

## Supplementary Material

sby189_suppl_Supplementary_Material
