# Supplementary material for: Using Machine Learning and Structural Neuroimaging to Detect First Episode Psychosis: Reconsidering the Evidence
Source: Schizophr Bull. 2019 Feb 27;46(1):17–26. doi: 10.1093/schbul/sby189 (PMC6942152; doi:10.1093/schbul/sby189)
Supplement: sby189_suppl_Supplementary_Material [file sby189_suppl_supplementary_material.docx]

**Supplementary material**

Contents

[1. eMethods 2](#_Toc1410668632)

[1.1. Participants 2](#_Toc345096372)

[1.1.1. Recruitment procedure and criteria 2](#_Toc290030144)

[1.1.2. Matching 4](#_Toc1301156510)

[1.2. MRI data acquisition 5](#_Toc771886332)

[1.3. MRI preprocessing 5](#_Toc1554538127)

[1.3.1. Voxel-based maps 5](#_Toc1542747438)

[1.3.2. Surface-based volume and cortical thickness 6](#_Toc463821401)

[1.4. eStatistical analysis 7](#_Toc1900284417)

[1.4.1. Group-level analysis 7](#_Toc2037128450)

[1.4.2. Multivariate pattern recognition analysis 7](#_Toc893963643)

[1.4.2.1. Dimensionality reduction: principal component analysis 7](#_Toc478345438)

[1.4.2.2. Feature scaling: Standardization 8](#_Toc748356210)

[1.4.2.3. Classifiers 8](#_Toc581816423)

[1.4.2.4. Performance measures 11](#_Toc1534599366)

1.4.2.5. Significance testing…………………………………………………………………………………...11

# 1.4.2.6. Effect of medication and psychotic symptoms…………………………………………................12

[2. eResults 12](#_Toc742551939)

[2.1. Group-level analyses 12](#_Toc1323445393)

[3. eDiscussion 21](#_Toc1974802194)

[3.1. Association between sample size and classification accuracy 21](#_Toc1277532988)

[3.2. Publication bias 21](#_Toc1404223000)

References……………………………………………………………………………………………………..22

eTables

[eTable 1. Sample size of each dataset. 4](#_Toc774774803)

[eTable 2. Image acquisition parameters for each site. 5](#_Toc1458261099)

[eTable 3. Parameters for tuning for DNN. 11](#_Toc1529511818)

[eTable 4. Group-level analysis: GMV. 13](#_Toc2106475836)

[eTable 5. Group-level analysis: VBCT. 14](#_Toc1141162874)

[eTable 6. Group-level analysis: surface-based regional volumes and cortical thickness. 15](#_Toc325542620)

[eTable 7. Group-level analysis controlling for age and gender: GMV. 16](#_Toc1835336428)

[eTable 8. Group-level analysis controlling for age and gender: VBCT. 16](#_Toc1134410382)

[eTable 9. Group-level analysis controlling for age and gender: surface-based regional volumes and cortical thickness. 17](#_Toc1829831445)

[eTable 10. Statistical significance for all classifiers. 19](#_Toc821002746)

eTable 11. Odds ratio, confidence interval and p-value for the effects of anti-psychotic medication and psychotic symptoms on predicted labels……………………………………………………………………. 20

# **1. eMethods**

## **1.1. Participants**

### **1.1.1. Recruitment procedure and criteria**

### Site1: Chengdu, China

First episode patients were recruited from the West China Hospital of Sichuan University in Chengdu (China), as part of a wider study of psychiatric disorders in China. Diagnosis and duration of illness were determined by the consensus of two clinical psychiatrists using the Structured Interview for the DSM-IV Axis I Disorder (SCID)^1^. At the time of scanning, all patients were medication-naïve. Healthy controls were recruited by poster advertisement and screened using the SCID-I to confirm the lifetime absence of psychiatric disorders, as well as interviewed and subsequently excluded if they had any known history of psychiatric illness in first-degree relatives. Participants were excluded if they met any of the following criteria: (i) history of drug or alcohol abuse, (ii) pregnancy, and (iii) any physical illness such as hepatitis, cardiovascular disease, or neurological disorder, as assessed by interview and review of medical records.

### Site 2: London, England

Participants were recruited from the South London and Maudsley Foundation Trust and scanned at the Institute of Psychiatry, Psychology and Neuroscience in London (England). Diagnosis of schizophrenia was formulated by an experienced psychiatrist using the ICD-10 criteria. Healthy controls were recruited through local advertisement from the same geographical areas as patients. A screening tool (Psychosis Screening Questionnaire^2^) was used to exclude the presence of psychotic symptomatology or a history of psychotic illness. Additional exclusion criteria for all participants included learning disabilities (based as an IQ < 70), current or past neurological illness, brain injury with loss of consciousness for more than 1 hour and suspected or confirmed pregnancy.

### Sites 3 and 4: Santander A and B, Spain

Data from Santander A and Santander B were acquired as part of the same large prospective longitudinal study on first episode psychosis in the region of Cantabria, although with two different scanners (eTable 2). Individuals with FEP were recruited from both inpatient units and community services throughout the entire region. Patients were included if they met the following criteria: 1) age 15–60 years; 2) DSM-IV criteria for a principal diagnosis of schizophrenia, schizophreniform disorder, schizoaffective disorder, brief reactive psychosis, or not otherwise specified psychosis; and 3) no prior treatment with antipsychotic medication or, if previously treated, a total lifetime of adequate antipsychotic treatment of less than 6 weeks. Patients with DSM-IV based diagnoses of mental retardation or substance dependence (except nicotine dependence) were excluded. Age and gender matched healthy controls were recruited from the community through advertisements and were screened for current or past history of psychiatric, mental retardation, neurological or general medical illness, including substance dependence and significant loss of consciousness, as determined by using an abbreviated version of the Comprehensive Assessment of Symptoms and History (CASH)^3^. Clinical records and family interview also confirmed the absence of psychosis in first-degree relatives.

### Site 5: Utrecht, The Netherlands

Inpatients and outpatients were identified by clinicians working in regional psychosis departments or academic centres and were included if they met the following criteria: 1) age range of 16 to 50 years; 2) a diagnosis of non‐affective psychotic disorder according to the DSM‐IV; 3) good command of the Dutch language; and 4) able and willing to give written informed consent. Controls were selected through a system of random mailings to addresses in the catchment areas of the cases and were included if the following criteria were met: 1) age range of 16 and 50 years, 2) no lifetime psychotic disorder, 3) no first-degree family member with a lifetime psychotic disorder, 4) good command of the Dutch language, and 5) able and willing to give written informed consent.

### 1.1.2. Matching

To maximize the use of the data made available, matching was carried out by taking the group with smallest sample size (first episode psychosis (FEP) or healthy controls (HC)) and randomly selecting participants from the other group according to age (+/- 5 years) and gender. For all sites, the FEP:HC matching ratio was 1:1, except for site 4 where the ratio was 2:1.

### eTable 1. Sample size of each dataset. We report the number of subjects available (top row), the number of subjects excluded after matching patients and controls for age and gender (middle row) and the final number of subjects included in the statistical analysis (bottom row).

|  | Site 1  Chengdu,  China | Site 2  London,  England | Site 3  Santander A,  Spain | Site 4  Santander B,  Spain | Site 5  Utrecht,  The Netherlands |
| --- | --- | --- | --- | --- | --- |
| Available | 330 | 204 | 257 | 223 | 225 |
| Excluded | 108 | 62 | 37 | 13 | 63 |
| Final | 222 | 142 | 220 | 210 | 162 |

## **1.2. MRI data acquisition**

|  | Site 1 | Site 2 | Site 3 | Site 4 | Site 5 |
| --- | --- | --- | --- | --- | --- |
| Field strength (T) | 3 | 3 | 3 | 1.5 | 1.5 |
| TR/TE (ms) | 8.5/3.4 | 6.9/2.8 | 8.2/3.7 | 24/5 | 30/4.6 |
| Slice thickness (mm) | 1 | 1.2 | 1 | 1.5 | 1.2 |
| Data matrix | 512x512x156 | 256x256x166 | 256x256x160 | 256x256x124 | 256x256x170 |
| Voxel size | 0.47x0.47x1 | 1.02x1.02x1.2 | 0.94x0.94x1 | 1.02x1.02x1.5 | 1x1x1.2 |

## **eTable 2.** Image acquisition parameters for each site.

## **1.3. MRI preprocessing**

After checking all T1-weighted images for scanner artefacts and gross anatomical abnormalities, images were preprocessed to extract three types of anatomical features: voxel-based grey matter volume, voxel-based cortical thickness and surface-based volumes and cortical thickness.

### 1.3.1. Voxel-based maps

Two different voxel-based features were extracted: grey matter volume and cortical thickness. Common to both features, images were first reoriented along the anterior-posterior commissure line and set the anterior commissure as the origin of the spatial coordinates to assist the normalization algorithm. Reoriented images were then segmented into grey matter (GM), white matter (WM) and cerebrospinal fluid (CSF) partitions as implemented in SPM12^4^ (http://www.fil.ion.ucl.ac.uk/spm).

### 1.3.1.1. Grey matter volume

The segmentation tissue maps for each site were pre-processed separately using the Diffeomorphic Anatomical Registration using the Exponentiated Lie algebra (DARTEL) toolbox^5^. This procedure warps the grey matter and white matter partitions into a new study-specific reference space representing an average of all the subjects included in the analysis, thus maximizing accuracy and sensitivity^6,7^. The warped grey matter partitions were then affine-transformed into MNI space. An additional modulation step was used to scale the grey matter probability values by the Jacobian determinants of the deformations, thereby ensuring that the total amount of grey matter in each voxel was conserved after registration^8.^ Finally, the GM probability maps were smoothed using a standard 8mm FWHM Gaussian kernel.

### 1.3.1.2. Cortical thickness

A voxel-based Laplacian method^9^, implemented as an SPM toolbox^10,11^, was used to create a voxel-based cortical thickness (VBCT) map for each subject using the GM, WM and CSF partitions generated in the segmentation step. Briefly, the resulting VBCT maps contained cortical thickness (CT) values within voxels identified as grey matter and zeros outside the cortex. Each VBCT map was warped into the corresponding site-specific DARTEL reference space. The warped images were then normalized to MNI space and smoothed with a 6 mm Gaussian kernel. The same warps, modulation and smoothing were also applied to a binary mask created from each original VBCT map. Subsequently the warped, scaled and smoothed VBCT maps were divided by the corresponding warped, scaled, and smoothed mask.

### 1.3.2. Surface-based volume and cortical thickness

FreeSurfer 5.3 (http://surfer.nmr.mgh.harvard.edu)^12^ was used to parcellate each participant’s raw brain image into subcortical and cortical regions according to the Desikan-Killiany atlas^13^ using the ‘recon-all’ command. FreeSurfer is a well-established automated procedure for imaging preprocessing and analysis which details have been extensively described elsewhere^14–16^. A total of 169 features were used, including 33 volumes of subcortical structures plus volume and thickness of 34 cortical regions per hemisphere (after removing white matter hypo-intensities, 5^th^ ventricle, optic chiasm and bilateral vessels and choroid plexus).

# 1.4. eStatistical analysis

## **1.4.1. Group-level analysis**

# 1.4.1.1. Grey matter volume and cortical thickness

Voxel-based morphometry (VBM) was used to calculate group-level differences in voxel-based grey matter volume and voxel-based cortical thickness between FEP and HC groups at each site. An independent-sample t-test was used with statistical inferences made at p<0.05 after family-wise error (FWE) correction for multiple comparisons and a minimum extent threshold of 5 voxels.

1.4.1.2. Surface-based regional volumes and cortical thickness

Surface-based regional volumes and cortical thickness were analysed with an independent-sample t-test as implemented in SPSS 24.0 using a statistical threshold of *p*<0.05 and additional Bonferroni correction for multiple comparisons.

All reported results (eResults, section 2.1) were obtained without covariates of no interest to ensure consistency between group- and individual-level statistical analyses. However, statistical analyses with age and gender as covariates were also carried out for completeness; this yielded identical results except for surface-based regional volumes and cortical thickness data (eTable 7-9).

# 1.4.2. Multivariate pattern recognition analysis

## 1.4.2.1. Dimensionality reduction: principal component analysis

Principal component analysis (PCA) is a well-established unsupervised method for feature reduction in neuroimaging. PCA reduces dimensionality by geometrically projecting the data into lower dimensions called principal components (PCs), with the aim of finding the best summary of the data using a limited number of PCs. PCA uses an orthogonal transformation to convert a set of observations of possibly correlated features into a set of values of uncorrelated features (PC). PCs are then ranked according to explained variance in descending order. A detailed description of PCA is given elsewhere^17,18^. In the present investigation, PCA was implemented within the CV framework; at each fold, dimensionality was reduced by 1) extracting the minimum number of principal components whilst retaining cumulative 90% of the variance from the data in the training set only, 2) projecting all grey matter/cortical thickness maps onto the resulting principal components and 3) using the resulting values for classification and 4) projecting the test data into the same components derived from the training set, and using the former for testing.

## 1.4.2.2. Feature scaling: Standardization

Standardization was performed by removing the mean and scaling to unit variance. This procedure was applied to each feature independently. Standardization is a common requirement for many ML methods, since algorithms might behave poorly if the individual features do not resemble normally distributed data. In addition, features with bigger scales might dominate the loss function of the training algorithms. To avoid “double dipping”, the statistics (mean and variance) were obtained using only the training set, and these same values were used in the standardization of test set.

## 1.4.2.3. Classifiers

K-nearest neighbour (KNN), logistic regression (LR) and support vector machine (SVM) were implemented using the Scikit-Learn library^19^ (sklearn) for python 3.5. Deep neural network (DNN) was implemented using Tensorflow v.1.4^20^ and Keras v.2.1^21^ libraries. The random seed was kept the same for all models to ensure the reproducibility of the results. This approach guaranteed that the starting weights and train/test split at each fold of the CV would remain the same within and between algorithms for the same site.

### 1.4.2.3.1. K-nearest neighbours

K-nearest neighbours (KNN) is a non-parametric method based on multivariate pairwise distance measures between data points. Once presented with unseen data, it calculates the Euclidean distance between this new data point and each of the surrounding neighbours. Classification is done by assigning the unseen data to the same class as the majority of its neighbours^22^. The optimal number of neighbours was tuned via grid search by testing 10 possible odd values ranging from 3 to 21 in increments of 2.

### 1.4.2.3.2. Logistic regression

Logistic regression (LR) was implemented via elastic net, a regularized regression that combines the regularizations L1 and L2 penalties of LASSO (Least Absolute Shrinkage and Selection Operator) and ridge regression, respectively. While the ridge penalty retains all variables and minimizes the impact of irrelevant features, the LASSO penalty discards unimportant variables^23^. Grid search was used to find the optimal relative contribution of each penalty via tuning of the hyperparameter l1_ratio as defined by sklearn from eleven possible values between 0 and 1 with increments of 0.1.

### 1.4.2.3.3. Support vector machine

Support vector machine (SVM) is a supervised machine learning technique that maps the input data into a feature space using a set of similarity functions known as kernels. In this feature space, the model finds the optimal separating hyperplane by finding the largest margin of separation between the two classes within the training set. Once the hyperplane is determined, it can be used to predict the class of new unseen observations^24,25^. In this study, a linear kernel was chosen to contrast with the characteristic non-linear approach of DL. The soft margin (C) parameter, that controls the trade-off between having zero training errors and allowing misclassifications, was tuned from a possible range of values (2^-5^, 2^-3^, ..., 2^13^, 2^15^) using grid search, i.e. all possible values in a given range were tested.

### 1.4.2.3.4. Deep neural network

Given its flexible architecture, deep learning can be used to build a variety of different neural networks^26^. Here we employed a deep neural network, with the components resulting from the PCA (for the VBM and VBCT data) or the regional volumes and cortical thickness as inputs; this architecture was chosen as it allowed for automated and non-biased optimization of the hyperparameters, which in turn helps prevent overfitting. Deep neural networks are multi-layered fully-connected networks where higher-level features are learned as a non-linear combination of lower-level features, thus allowing the extraction of complex and abstract patterns from the data. Once the model learns these higher-level features, it can determine a separation surface to classify the different classes^26,27^. The performance of DNN models relies on the specification of several architectural and learning hyperparameters. To prevent bias, the number of layers, number of units, optimizer, learning rate, decay, activation function and epoch and were optimized using random search as implemented by sklearn. To decrease the chances of overfitting, two additional parameters were also included at each layer: i) L2 regularizer, which penalizes high weights^28^ and ii) dropout, where randomly selected neurons are ignored during training^29^. Each layer was initialized via Glorot (also known as Xavier) initialization (normal distribution)^30^. In the output layer, the classification was performed by a softmax function. Training was carried out using a mini-batch with 8 training samples for VBM and VBCT, and 128 for surface-based volumes and cortical thickness. DNN models were optimized via random search due to the high number of parameters to test: at each fold, 500 different combinations of randomly selected values for each parameter were tested. eTable 3, shows all the possible values for each parameter.

| Parameter | Values |
| --- | --- |
| Number of layers | 2, 3, 4, 5 |
| Number of units | 10, 20, 50, 75, 100, 150 |
| Activation function | ReLU, Leaky ReLU |
| Learning rate | 0.001, 0.005, 0.01, 0.1, 0.2 |
| Learning rate decay | 10^-6^, 10^-5^, 10^-4^, 10^-3^ |
| Epochs | 50, 100, 150 |
| Optimizer | Stochastic gradient descent (SGD), Adam |
| Momentum | 0.99, 0.9, 0.95 |
| L2 coefficient | 10^-5^, 10^-4^, 10^-3^, 10^-2^ |
| Drop-out rate | 0.2, 0.5, 0.7 |

### **eTable 3.** Parameters for tuning for DNN

##

## 1.4.2.4. Performance measures

Performance metrics were calculated according to the below formulas:

Sensitivity = TP/(TP+FN)

Specificity = TN/(TN+FP)

Balanced accuracy = (Sensitivity + Specificity)/2

1.4.2.5. Significance testing

The balanced accuracy of each classifier was tested for significance using permutation testing, whereby subjects were randomly assigned to one of the classes (patients/control), so that the labels no longer match the data in any meaningful way, and the 10-fold CV cycle repeated 1000 times. This resulted in a distribution of accuracies reflecting the null hypothesis that the classifier did not exceed chance. The number of times the classifier’s performance was greater than or equal to the true accuracy was divided by 1000 to determine a p-value. A p-value lower than 0.05 was considered statically significant.

# 1.4.2.6. Effect of medication and psychotic symptoms

# To examine whether anti-psychotic medication and psychotic symptoms contributed to the classifiers’ performance, chlorpromazine equivalents and positive and negative psychotic symptoms were regressed against the predicted labels using a logistic regression as implemented by the Logit function from the statsmodel python library. Because all patients from site 1 were anti-psychotic naïve, the investigation of the effects of medication was limited to sites 2, 3, 4 and 5. The size of the effects of medication and psychotic symptoms was measured in terms of odds ratio (OR) and respective 95% confidence interval (CI). The statistical significance threshold was set to 0.05.

# 2. eResults

# 2.1. Group-level analyses

No significant GMV decreases in FEP relative HC were found at any site. In contrast, GMV increases were detected in the bilateral thalamus at site 3; in the left putamen and the right pallidum at site 4; and in the right putamen at site 5. No significant increased or decreased VBCT was observed in FEP compared to HC at any site, except for site 1 in which FEP showed increased thickness in the left fusiform gyrus and left superior frontal gyrus. Significant differences in surface-based regional volumes and cortical thickness between FEP and HC were found for sites 3 and 4. At site 3, patients showed smaller right hippocampus volume as well as a reduced thickness of the inferior parietal lobe; whereas at site 4 patients showed a significant cortical thinning in the left inferior temporal gyrus, pars opercularis and rostral middle frontal gyrus, as well as a larger 3^rd^ ventricle. These results are presented in detail in eTables 4-7.

**eTable 4.** Group-level analysis: GMV.

| Region | Peak MNI Coordinates  (x,y,z) | Cluster size  (No. of Voxels) | z | *p* |
| --- | --- | --- | --- | --- |
| FEP > HC |  |  |  |  |
| Site 3 |  |  |  |  |
| Left thalamus | -16,-28,12 | 37 | 5.5 | .006 |
| Right thalamus | 16,-24,14 | 17 | 4.7 | .014 |
| Site 4 |  |  |  |  |
| Left putamen | -30,-12,-4 | 118 | 5.3 | .001 |
| Right pallidum | 20,-10,-4 | 17 | 4.6 | .014 |
| Site 5 |  |  |  |  |
| Right putamen | 30,-8,-4 | 85 | 5.3 | .001 |

**eFigure 1.** Regions with increased GMV in FEP relative to controls in site 3 (A,) 4 (B) and 5 (C).


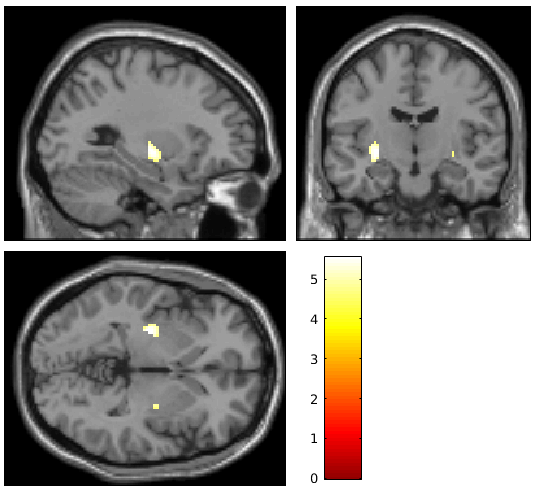


B


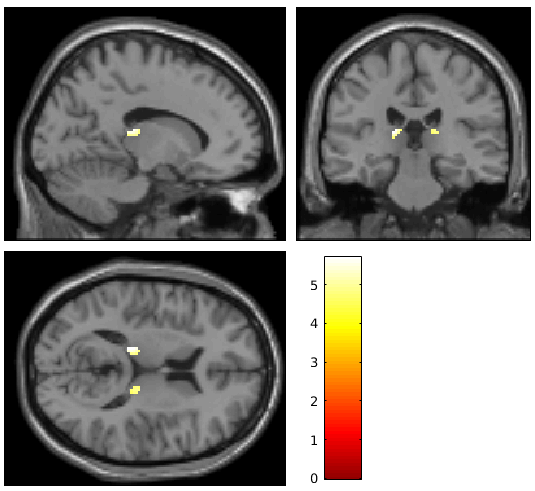


A


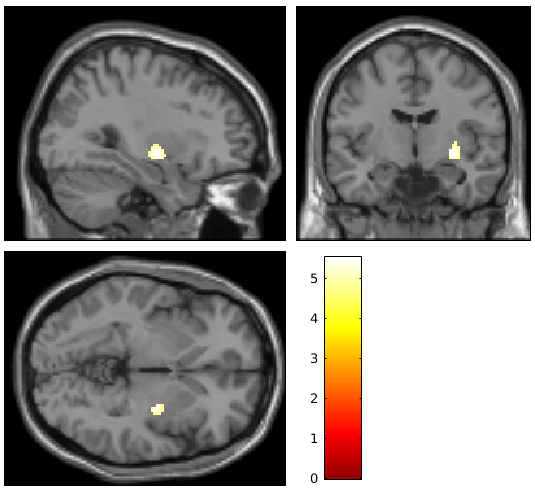


C

| Region | Peak MNI Coordinates  (x,y,z) | Cluster size  (No. of Voxels) | z | *p* |
| --- | --- | --- | --- | --- |
| FEP > HC |  |  |  |  |
| Site 1 |  |  |  |  |
| Left fusiform gyrus | -30,-10,-36 | 11 | 4.7 | .012 |
| Left superior frontal gyrus | -10,58,36 | 11 | 4.5 | .012 |

**eTable 5.** Group-level analysis: VBCT.

**
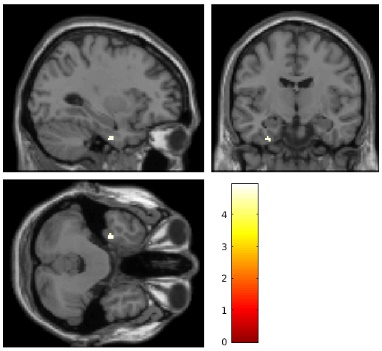
eFigure 2.** Cortical region with increased GMV in FEP relative to controls in site 1.

## **eTable 6.** Group-level analysis: surface-based regional volumes and cortical thickness.

| Region | *t* | *p* |
| --- | --- | --- |
| FEP < HC |  |  |
| Site 3 |  |  |
| Right hippocampus | 4.3 | <.001 |
| Left inferior parietal (thickness) | 3.9 | <.001 |
| Site 4 |  |  |
| Left inferior temporal gyrus (thickness) | 3.6 | <.001 |
| Left pars opercularis (thickness) | 4.0 | <.001 |
| Left rostral middle frontal gyrus (thickness) | 4.8 | <.001 |
| FEP > HC |  |  |
| Site 4 |  |  |
| Third ventricle | -4.1 | <.001 |

##

## **eTable 7.** Group-level analysis controlling for age and gender: GMV.

| Region | Peak MNI Coordinates  (x,y,z) | Cluster size  (No. of Voxels) | z | *p* |
| --- | --- | --- | --- | --- |
| FEP > HC |  |  |  |  |
| Site 3 |  |  |  |  |
| Left thalamus | -16,-28,12 | 40 | 5.6 | .006 |
| Right thalamus | 16,-24,14 | 19 | 4.8 | .014 |
| Site 4 |  |  |  |  |
| Left putamen | -30,-14,-2 | 110 | 5.1 | .001 |
| Right pallidum | 28,-10,-4 | 17 | 4.6 | .014 |
| Site 5 |  |  |  |  |
| Right putamen | 30,-8,-6 | 70 | 5.2 | .002 |

| Region | Peak MNI Coordinates  (x,y,z) | Cluster size  (No. of Voxels) | z | *p* |
| --- | --- | --- | --- | --- |
| FEP > HC |  |  |  |  |
| Site 1 |  |  |  |  |
| Left fusiform gyrus | -30,-10,-36 | 16 | 5.0 | .008 |
| Left superior frontal gyrus | -10,58,36 | 11 | 4.6 | .012 |

**eTable 8.** Group-level analysis controlling for age and gender: VBCT.

## **eTable 9.** Group-level analysis controlling for age and gender: surface-based regional volumes and cortical thickness.

| Region | *F* | *p* |
| --- | --- | --- |
| FEP < HC |  |  |
| Site 3 |  |  |
| Right hippocampus | 22.4 | <.001 |
| Left inferior parietal gyrus (thickness) | 20.7 | <.001 |
| Left precuneos (thickness) | 15.3 | <.001 |
| Left superior frontal gyrus (thickness) | 12.8 | <.001 |
| Left supramarginal gyrus (thickness) | 17.2 | <.001 |
| Site 4 |  |  |
| Left parsopercularis (thickness) | 15.6 | <.001 |
| Left rostral middle frontal gyrus (thickness) | 21.9 | <.001 |
| Site 5 |  |  |
| Left hippocampus | 15.7 | <.001 |
| FEP > HC |  |  |
| Site 3 |  |  |
| Left lateral ventricle | 14.8 | <.001 |
| Site 4 |  |  |
| Third ventricle | 17.2 | <.001 |

**eFigure 3.** Balanced accuracies and standard deviations of the different algorithms and feature sets for each site.


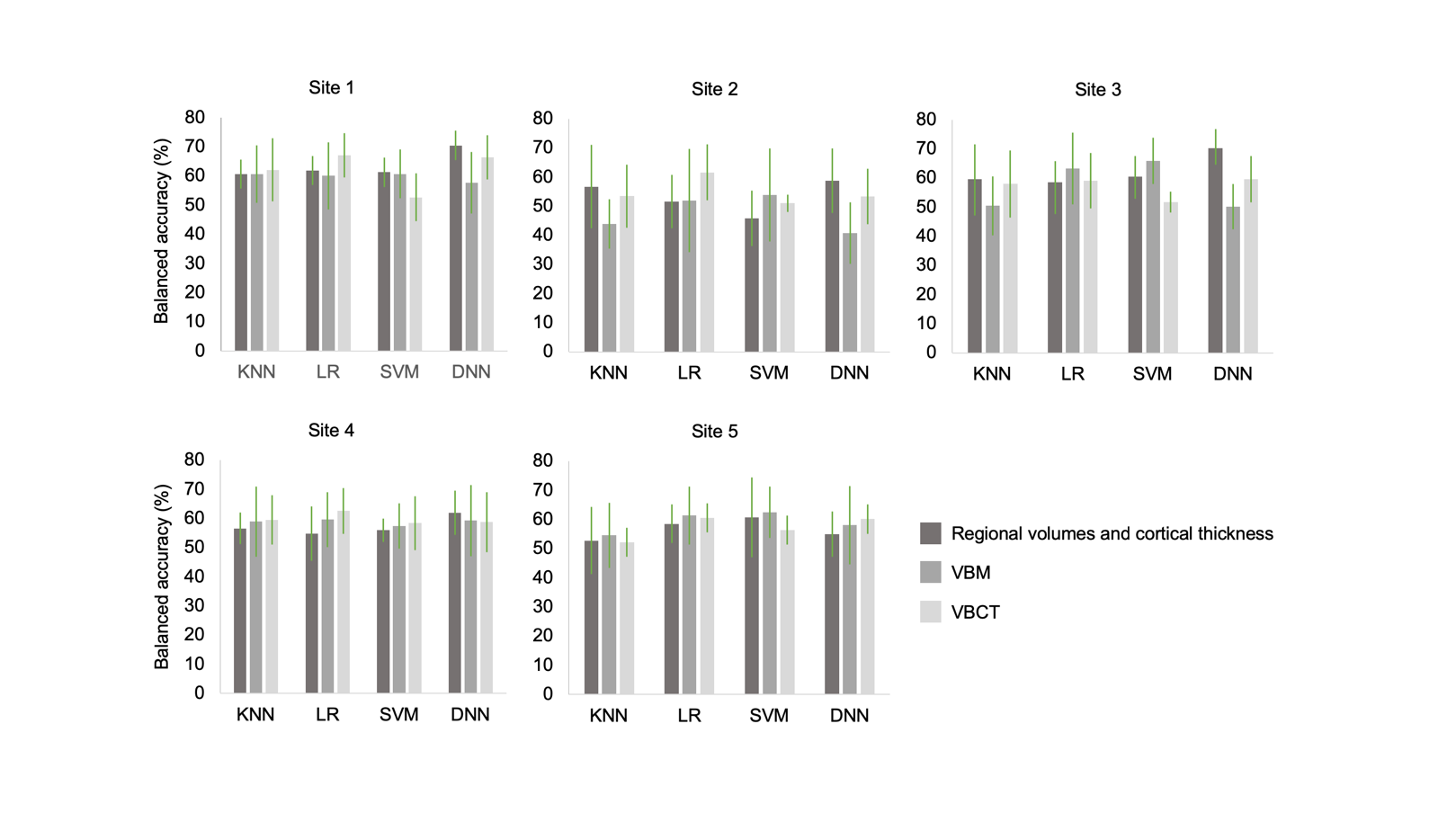


KNN: k-nearest neighbours; LR: logistic regression; SVM: support vector machines; DNN: deep neural network; VBM: voxel-based morphometry; VBCT: voxel-based cortical thickness.

## **eTable 10**. Statistical significance for all classifiers.

|  |  | Surface-based regional volumes and cortical thickness | VBM | VBCT |
| --- | --- | --- | --- | --- |
| Site 1  Chengdu  China | KNN | **.003** | **.001** | **.002** |
|  | LR | **.003** | **.003** | **.001** |
|  | SVM | **.003** | **.004** | **.013** |
|  | DNN | **.001** | **.008** | **.001** |
| Site 2  London  England | KNN | .083 | .891 | .200 |
|  | LR | .381 | .346 | **.009** |
|  | SVM | .757 | .207 | .450 |
|  | DNN | **.014** | .593 | .265 |
| Site 3  Santander A  Spain | KNN | **.004** | .444 | **.011** |
|  | LR | **.021** | **.002** | **.013** |
|  | SVM | **.005** | **.001** | **.036** |
|  | DNN | **.001** | .448 | **.010** |
| Site 4  Santander B  Spain | KNN | **.041** | **.003** | **.028** |
|  | LR | .129 | **.012** | **.001** |
|  | SVM | .081 | **.032** | **.030** |
|  | DNN | **.001** | **.014** | **.002** |
| Site 5  Utrecht  The Netherlands | KNN | .237 | .163 | .262 |
|  | LR | **.033** | **.003** | **.007** |
|  | SVM | **.007** | **.004** | .408 |
|  | DNN | .108 | **.010** | **.008** |

eTable 11. Odds ratio, confidence interval and p-value for the effects of anti-psychotic medication and psychotic symptoms on predicted labels.

|  | | Surface-based regional volumes and cortical thickness | | | VBM | | | VBCT | | |
| --- | --- | --- | --- | --- | --- | --- | --- | --- | --- | --- |
|  |  | Anti-psychotic medication | Positive symptoms | Negative symptoms | Anti-psychotic medication | Positive symptoms | Negative symptoms | Anti-psychotic medication | Positive symptoms | Negative symptoms |
| SITE 1 | KNN | - | 0.99 [0.97-1.09], .656 | 0.98 [0.97-1.09], .575 | - | 1.03 [0.97-1.09], .333 | 1.0 [0.94-1.04], .587 | - | 1.03 [0.97-1.1], .334 | 1.0 [0.94-1.05], .768 |
|  | LR | - | 1.0 [0.97-1.09], .896 | 1.03 [0.97-1.09], .264 | - | 0.97 [0.91-1.03], .276 | 1.0 [0.97-1.07], .516 | - | 1.01 [0.95-1.07], .783 | 1.0 [0.99-1.1], .117 |
|  | SVM | - | 1.0 [0.97-1.09], .896 | 1.03 [0.97-1.09], .264 | - | 0.99 [0.93-1.05], .754 | 1.0 [0.93-1.03], .477 | - | 0.96 [0.87-1.06], .464 | 1.0 [0.89-1.08], .676 |
|  | DL | - | 0.97 [0.97-1.09], .378 | 1.03 [0.97-1.09], .351 | - | 0.99 [0.93-1.05], .724 | 1.0 [0.95-1.05], .957 | - | 1.05 [0.98-1.11], .142 | 1.0 [0.95-1.06], .905 |
| SITE 2 | KNN | **1.0 [1.0-1.01], .038** | 1.0 [0.91-1.11], .971 | 1.0 [0.89-1.05], .447 | 1.0 [1.0-1.01], .292 | 0.91 [0.81-1.03], .135 | 1.0 [0.9-1.07], .641 | 1.0 [1.0-1.0], .875 | 1.01 [0.9-1.13], .929 | 1.0 [0.92-1.1], .864 |
|  | LR | 1.0 [1.0-1.0], .990 | 1.02 [0.93-1.12], .694 | 1.0 [0.9-1.05], .433 | 1.0 [1.0-1.0], .683 | 0.99 [0.9-1.09], .887 | 1.0 [0.94-1.09], .740 | 1.0 [1.0-1.0], .463 | 0.93 [0.83-1.05], .262 | 1.0 [0.94-1.14], .468 |
|  | SVM | 1.0 [1.0-1.0], .680 | **0.86 [0.76-0.97], .011** | 1.0 [0.92-1.09], .889 | 1.0 [1.0-1.01], .212 | 0.96 [0.87-1.07], .475 | 1.0 [0.9-1.06], .535 | 1.0 [0.9-1.12], .949 | 1.0 [1.0-1.0], .775 | 1.0 [0.95-1.1], .673 |
|  | DL | 1.0 [1.0-1.0], .956 | 0.95 [0.85-1.05], .289 | 1.0 [0.87-1.03], .192 | 1.0 [1.0-1.01], .275 | 1.04 [0.94-1.14], .485 | 1.0 [0.88-1.03], .241 | 1.0 [1.0-1.0], .344 | 0.96 [0.86-1.08], .518 | 1.0 [0.89-1.06], .503 |
| SITE 3 | KNN | 1.0 [1.0-1.0], .522 | 0.95 [0.86-1.04], .253 | 1.0 [0.96-1.11], .456 | 1.0 [1.0-1.0], .916 | 1.06 [0.96-1.18], .236 | 1.0 [0.99-1.16], .092 | 1.0 [1.0-1.0], .741 | 0.96 [0.88-1.04], .268 | 1.0 [0.96-1.09], .515 |
|  | LR | 1.0 [1.0-1.0], .516 | 1.01 [0.92-1.11], .799 | 1.0 [0.91-1.05], .561 | 1.0 [1.0-1.0], .285 | 0.97 [0.88-1.07], .548 | **1.0 [1.0-1.2], .049** | 1.0 [1.0-1.0], .098 | 1.0 [0.92-1.08], .942 | 1.0 [0.94-1.07], .930 |
|  | SVM | 1.0 [1.0-1.0], .188 | 1.05 [0.95-1.15], .357 | 1.0 [0.89-1.04], .320 | 1.0 [1.0-1.0], .560 | 0.96 [0.87-1.05], .366 | 1.0 [0.95-1.12], .416 | 1.0 [1.0-1.0], .620 | 0.94 [0.82-1.08], .400 | 1.0 [0.89-1.1], .797 |
|  | DL | 1.0 [1.0-1.0], .277 | 1.01 [0.92-1.11], .825 | 1.0 [0.91-1.06], .593 | 1.0 [1.0-1.0], .926 | 0.96 [0.88-1.06], .414 | 1.0 [0.96-1.11], .459 | 1.0 [1.0-1.0], .817 | 1.01 [0.93-1.09], .815 | 1.0 [0.94-1.08], .806 |
| SITE 4 | KNN | 1.0 [1.0-1.0], .661 | 0.92 [0.79-1.09], .343 | 1.0 [0.78-1.02], .086 | 1.0 [1.0-1.0], .380 | 1.01 [0.92-1.12], .778 | 1.0 [0.93-1.11], .697 | 1.0 [1.0-1.0], .661 | 1.0 [1.0-1.0], .735 | 1.0 2 [0.91-1.12], .534 |
|  | LR | 1.0 [1.0-1.0], .389 | 1.04 [0.94-1.15], .439 | 1.0 [0.84-1.0], .050 | 1.0 [1.0-1.0], .769 | 1.08 [0.99-1.19], .089 | 1.0 [0.89-1.04], .328 | 1.03 [0.95-1.1], .463 | 1.0 [1.0-1.0], .385 | 1.0 [1.0-1.0], .638 |
|  | SVM | 1.0 [1.0-1.0], .584 | 1.01 [0.92-1.1], .894 | **1.0 [0.85-0.99], .033** | 1.0 [1.0-1.0], .126 | 1.04 [0.94-1.14], .482 | 1.0 [0.88-1.05], .355 | 1.0 [1.0-1.0], .884 | 1.0 [1.0-1.0], .472 | 0.96 [0.89-1.03], .264 |
|  | DL | 1.0 [1.0-1.0], .621 | 1.04 [0.94-1.15], .493 | 1.0 [0.85-1.01], .072 | 1.0 [1.0-1.0], .572 | 0.95 [0.85-1.06], .356 | **1.0 [0.82-0.99], .034** | 1.0 [1.0-1.0], .521 | 1.02 [0.89-1.14], .573 | 1.0 [1.0-1.0], .647 |
| SITE 5 | KNN | 1.0 [0.99-1.0], .207 | 0.99 [0.89-1.1], .847 | 1.0 [0.92-1.08], .947 | 1.0 [1.0-1.0], .375 | 1.04 [0.93-1.16], .464 | 1.0 [0.91-1.08], .828 | 1.0 [1.0-1.0], .489 | 0.96 [0.84-1.09], .535 | 1.0 [0.98-1.18], .128 |
|  | LR | 1.0 [1.0-1.0], .195 | **0.85 [0.75-0.97], .017** | 1.0 [0.87-1.04], .267 | 1.0 [1.0-1.0], .306 | 1.06 [0.96-1.18], .260 | 1.0 [0.91-1.06], .615 | 1.0 [1.0-1.0], .475 | 0.94 [0.83-1.07], .365 | 1.0 [0.93-1.1], .836 |
|  | SVM | 1.0 [1.0-1.0], .313 | 0.99 [0.89-1.1], .795 | 1.0 [0.85-1.02], .108 | 1.0 [0.99-1.0], .105 | 1.06 [0.94-1.18], .338 | 1.0 [0.86-1.02], .160 | 1.0 [1.0-1.0], .400 | 1.03 [0.91-1.16], .647 | 1.0 [0.87-1.03], .226 |
|  | DL | 1.0 [0.99-1.0], .112 | 0.98 [0.87-1.1], .706 | **1.0 [0.81-0.99], .027** | 1.0 [1.0-1.0], .318 | 0.96 [0.86-1.06], .408 | 1.0 [0.98-1.15], .169 | 1.0 [1.0-1.01], .097 | 0.95 [0.83-1.08], .443 | **1.0 [0.82-1.0], .048** |

OR: odds ratio; CI: confidence interval; KNN: k-nearest neighbour; LR: logistic regression; SVM: support vector machine; DNN: deep neural network

# 3. eDiscussion

## 3.1. Association between sample size and classification accuracy

Accuracy and sample sizes from existing studies using ML and sMRI were extracted as follows:

- - up until and including 2013, this information was taken from the latest meta-analysis Kambeitz et al^31^;
- - from 2014 to 2016 this information was taken from the review Arbabshirani et al^32;^
- - seven further subsequent studies were identified: Pinaya et al^33^; Salvador et al^34^; Winterburn et al^35^; Xiao et al^36^; Rozycki et al^37^; Dluhoš et al^38^ and de Moura et al^39^. Xiao et al^36^ was excluded as it was a clear outlier (see Figure 3a).

Pearson’s correlation was used to test for the association between all sample sizes and accuracies. The same studies were used for Figure 1a.

## 3.2. Publication bias

Sample size, true positives, false positives, true negatives and false negative scores from each study as well as the overall main effect of ML-sMRI studies in psychosis (established schizophrenia and FEP combined) were extracted from Kambeitz et al^31^. Publication bias was assessed using the same procure as in Kambeitz et al^31^ which in turn was based on recommendations for diagnostic classification studies described in Deeks et al^40^. Briefly, a measure of sample size and effect size were calculated as follows:

- Effective sample size (ESS) was calculated from the patients and control groups sample size using the formula:


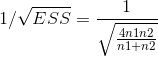


- lnDOR (diagnostic odds ratio) was calculated using the following formula:

#
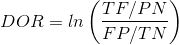


The resulting funnel plot was tested for asymmetry through a regression analysis weighted by ESS as implemented in R statistical programming language version 1.1.453 (R Core Team, 2016).

**References**

1. First MB, Gibbon M, Spitzer RL WJ. *Structured Clinical Interview for DSM-IV Axis II Personality Disorders*. American Psychiatric Press: Washington; 1997.

2. Bebbington P, Nayani T. The psychosis screening questionnaire. *Int J Methods Psychiatr Res*. 1995;5:11-19.

3. Andreasen NC, Flaum M, Arndt S. The Comprehensive Assessment of Symptoms and History (CASH). *Arch Gen Psychiatry*. 1992;49(8):615. doi:10.1001/archpsyc.1992.01820080023004

4. Ashburner J, Friston KJ. Unified segmentation. *Neuroimage*. 2005;26(3):839-851. doi:10.1016/j.neuroimage.2005.02.018

5. Ashburner J. A fast diffeomorphic image registration algorithm. *Neuroimage*. 2007;38(1):95-113. doi:10.1016/j.neuroimage.2007.07.007

6. Yassa M, Stark C. A quantitative evaluation of cross-participant registration techniques for MRI studies of the medial temporal lobe. *Neuroimage*. 2009;44(2):319-327. doi:10.1016/j.neuroimage.2008.09.016

7. Scarpazza C, Tognin S, Frisciata S, Sartori G, Mechelli A. False positive rates in Voxel-based Morphometry studies of the human brain: Should we be worried? *Neurosci Biobehav Rev*. 2015;52:49-55. doi:10.1016/j.neubiorev.2015.02.008

8. Mechelli A, Price C, Friston K, Ashburner J. Voxel-Based Morphometry of the Human Brain: Methods and Applications. *Curr Med Imaging Rev*. 2005;1(2):105-113. doi:10.2174/1573405054038726

9. Jones SE, Buchbinder BR, Aharon I. Three-dimensional mapping of cortical thickness using Laplace’s equation. *Hum Brain Mapp*. 2000;11(1):12-32.

10. Hutton C, De Vita E, Ashburner J, Deichmann R, Turner R. Voxel-based cortical thickness measurements in MRI. *Neuroimage*. 2008;40(4):1701-1710. doi:10.1016/j.neuroimage.2008.01.027

11. Hutton C, Draganski B, Ashburner J, Weiskopf N. A comparison between voxel-based cortical thickness and voxel-based morphometry in normal aging. *Neuroimage*. 2009;48(2):371-380. doi:10.1016/j.neuroimage.2009.06.043

12. Fischl B. FreeSurfer. *Neuroimage*. 2012;62(2):774-781. doi:10.1016/j.neuroimage.2012.01.021

13. Desikan RS, Ségonne F, Fischl B, et al. An automated labeling system for subdividing the human cerebral cortex on MRI scans into gyral based regions of interest. *Neuroimage*. 2006;31(3):968-980. doi:10.1016/j.neuroimage.2006.01.021

14. Dale AM, Fischl B, Sereno MI. Cortical Surface-Based Analysis. *Neuroimage*. 1999;9(2):179-194. doi:10.1006/nimg.1998.0395

15. Fischl B, Salat DH, Busa E, et al. Whole brain segmentation: automated labeling of neuroanatomical structures in the human brain. *Neuron*. 2002;33(3):341-355. doi:10.1016/S0896-6273(02)00569-X

16. Fischl B, Salat DH, van der Kouwe AJW, et al. Sequence-independent segmentation of magnetic resonance images. *Neuroimage*. 2004;23:S69-S84. doi:10.1016/j.neuroimage.2004.07.016

17. Jolliffe I. *Principal Component Analysis*. Springer, Berlin; 2002.

18. Lever J, Krzywinski M, Altman N. Points of Significance: Principal component analysis. *Nat Methods*. 2017;14(7):641-642. doi:10.1038/nmeth.4346

19. Pedregosa F, Varoquaux G, Gramfort A, et al. Scikit-learn: Machine Learning in Python. *J Mach Learn Res*. 2011;12(Oct):2825-2830.

20. Abadi M, Chu A, Goodfellow I, et al. Deep Learning with Differential Privacy. In: *Proceedings of the 2016 ACM SIGSAC Conference on Computer and Communications Security.* 2016:308-318. doi:10.1145/2976749.2978318

21. Chollet F, others. Keras. 2015.

22. Altman NS. An Introduction to Kernel and Nearest-Neighbor Nonparametric Regression. *Am Stat*. 1992;46(3):175-185. doi:10.1080/00031305.1992.10475879

23. Zou H, Hastie T. Regularization and variable selection via the elastic net. *J R Stat Soc Ser B (Statistical Methodol)*. 2005;67(2):301-320.

24. Pereira F, Mitchell T. Machine learning classifiers and fMRI : a tutorial overview. 2008:1-21. doi: 10.1016/j.neuroimage.2008.11.007

25. Vapnik V. *The Nature of Statistical Learning Theory*. Springer; 1995.

26. LeCun Y, Bengio Y, Hinton G. Deep learning. *Nature*. 2015;521(7553):436-444. doi:10.1038/nature14539

27. Vieira S, Pinaya WHL, Mechelli A. Using deep learning to investigate the neuroimaging correlates of psychiatric and neurological disorders: Methods and applications. *Neurosci Biobehav Rev*. 2017;74:58-75. doi:10.1016/j.neubiorev.2017.01.002

28. Krogh A, Hertz JA. A Simple Weight Decay Can Improve Generalization. In: Lippman DS, Moody JE, Touretzky DS, eds. Advances in Neural Information Processing Systems, Vol. 4. Morgan Kaufmann; 1992:950-957.

29. Srivastava N, Hinton G, Krizhevsky A, Sutskever I, Salakhutdinov R. Dropout: A Simple Way to Prevent Neural Networks from Overfitting. *J Mach Learn Res*. 2014;15:1929-1958.

30. Glorot X, Bengio Y. Understanding the difficulty of training deep feedforward neural networks. In: *Proceedings of the Thirteenth International Conference on Artificial Intelligence and Statistics*. 2010:249-256.

31. Kambeitz J, Kambeitz-Ilankovic L, Leucht S, et al. Detecting neuroimaging biomarkers for schizophrenia: a meta-analysis of multivariate pattern recognition studies. *Neuropsychopharmacology*. 2015;40(7):1742-1751. doi:10.1038/npp.2015.22

32. Arbabshirani MR, Plis S, Sui J, Calhoun VD. Single subject prediction of brain disorders in neuroimaging: Promises and pitfalls. *Neuroimage*. 2017;145:137-165. doi:10.1016/j.neuroimage.2016.02.079

33. Pinaya WHL, Gadelha A, Doyle OM, et al. Using deep belief network modelling to characterize differences in brain morphometry in schizophrenia. *Sci Rep*. 2016;6(38897). doi:10.1038/srep38897

34. Salvador R, Radua J, Canales-Rodríguez EJ, et al. Evaluation of machine learning algorithms and structural features for optimal MRI-based diagnostic prediction in psychosis. *PLoS One*. 2017;12(4):e0175683. doi:10.1371/journal.pone.0175683

35. Winterburn JL, Voineskos AN, Devenyi GA, et al. Can we accurately classify schizophrenia patients from healthy controls using magnetic resonance imaging and machine learning? A multi-method and multi-dataset study. *Schizophr Res*. December 2017. doi:10.1016/ j.schres.2017.11.038

36. Xiao Y, Yan Z, Zhao Y, et al. Support vector machine-based classification of first episode drug-naïve schizophrenia patients and healthy controls using structural MRI. *Schizophr Res*. December 2017. doi:10.1016/j.schres.2017.11.037

37. Rozycki M, Satterthwaite TD, Koutsouleris N, et al. Multisite Machine Learning Analysis Provides a Robust Structural Imaging Signature of Schizophrenia Detectable Across Diverse Patient Populations and Within Individuals. *Schizophr Bull*. 2018;44(5):1035-1044. doi:10.1093/schbul/sbx137

38. Dluhoš P, Schwarz D, Cahn W, et al. Multi-center Machine Learning in Imaging Psychiatry: A Meta-Model Approach. *Neuroimage*. 2017;155:10-24. doi:10.1016/j.neuroimage.2017.03.027

39. de Moura AM, Pinaya WHL, Gadelha A, et al. Investigating brain structural patterns in first episode psychosis and schizophrenia using MRI and a machine learning approach. *Psychiatry Res Neuroimaging*. 2018;275:14-20. doi:10.1016/j.pscychresns.2018.03.003

40. Deeks JJ, Macaskill P, Irwig L. The performance of tests of publication bias and other sample size effects in systematic reviews of diagnostic test accuracy was assessed. *J Clin Epidemiol*. 2005;58(9):882-893. doi:10.1016/j.jclinepi.2005.01.016
